# Kimchi improves irritable bowel syndrome: results of a randomized, double-blind placebo-controlled study

**DOI:** 10.29219/fnr.v66.8268

**Published:** 2022-05-23

**Authors:** Hee-Young Kim, Eui-Seong Park, Young Sik Choi, Seun Ja Park, Jae Hyun Kim, Hee Kyung Chang, Kun-Young Park

**Affiliations:** 1 Korean Medicine Research Center for Healthy Aging, Pusan National University, Yangsan, Gyeongsangnam-do, Republic of Korea; 2 Yuhan Care R&D Center, Yongin, Gyeonggi-do, Republic of Korea; 3 Department of Internal Medicine, Kosin University Gospel Hospital, Kosin University College of Medicine, Busan, Republic of Korea; 4 Department of Pathology, Kosin University Gospel Hospital, Kosin University College of Medicine, Busan, Republic of Korea; 5 Department of Food Science and Biotechnology, Cha University, Seongnam, Gyeonggi-do, Republic of Korea; 6 Chongqing Collaborative Innovation Center for Functional Food, Chongqing University of Education, Chongqing, China

**Keywords:** kimchi, irritable bowel syndrome (IBS), lactic acid bacteria (LAB), Lactobacillus plantarum nF1, microbiome, gut, clinical study

## Abstract

**Background:**

Irritable bowel syndrome (IBS) can be caused by abnormal bowel movements, altered brain-gut axis, gut microbiota change, and low levels of inflammation or immune activation. The intake of food containing much fiber and lactic acid bacteria (LABs) can alleviate IBS.

**Objective:**

This study was undertaken to confirm the alleviative effect of kimchi on symptoms of IBS.

**Design:**

Three types of kimchi (standard kimchi, SK; dead nano-sized *Lactobacillus plantarum* nF1 (nLp) added to standard kimchi, nLpSK; or functional kimchi, FK) were given to 30 individuals in each of three groups, that is, the SK group (*n* = 30), the nLpSK group (*n* = 30), or the FK group (*n* = 30) at 210 g a day for 12 weeks. Food intake records, serum levels of inflammatory factors, fecal levels of harmful enzymes, and microbiome changes were investigated over the 12-week study period.

**Results:**

After intervention, dietary fiber intake was increased in all groups. Typical IBS symptoms (abdominal pain or inconvenience, desperation, incomplete evacuation, and bloating), defecation time, and stool type were also improved. In serum, all groups showed reductions in tumor necrosis factor (TNF)-α (*P* < 0.001) levels. In addition, serum IL-4 (*P* < 0.001), IL-10 (*P* < 0.001), and IL-12 (*P* < 0.01) were significantly reduced in the nLpSK and FK groups, and serum monocyte chemotactic protein (MCP)-1 (*P* < 0.05) was significantly reduced in the nLpSK group. Furthermore, activities of fecal β-glucosidase and β-glucuronidase were significantly decreased in all three groups, and these reductions were greatest in the nLpSK group. Gut microbiome analysis showed that kimchi consumption increased Firmicutes populations at the expense of Bacteroidetes and Tenericutes populations. In addition, the *Bifidobacterium adolescentis* population increased significantly in the FK group (*P* = 0.026).

**Conclusion:**

Kimchi intake helps alleviate IBS by increasing dietary fiber intake and reducing serum inflammatory cytokine levels and harmful fecal enzyme activities. Notably, nLp improved the immune system, and several functional ingredients in FK promoted the growth of *Bifidobacterium adolescentis* in gut.

## Popular scientific summary

Irritable bowel syndrome (IBS) is characterized by an abnormal bowel condition and chronic mild inflammation. In this study, the intake of kimchi effectively reduced IBS symptoms and inflammation. Functional sub-ingredients (dead nano-sized *Lactobacillus plantarum* nF1, *Lactobacillus plantarum* PNU, mistletoe, etc.) added to kimchi enhanced the ameliorative effect of kimchi on IBS by improving immune functions and gut condition.

These days, gastrointestinal (GI) disorders such as irritable bowel syndrome (IBS), inflammatory bowel disease (IBD), and colorectal cancer are all too common. IBS is a type of functional GI disorder and the most common disease of the digestive system (1). IBS is not a painful disease, but it produces chronic abdominal symptoms such as abdominal discomfort, pain, incomplete evacuation, desperation, bloating, diarrhea, and constipation (1) and, thus, can diminish quality of life. According to several reports, the worldwide prevalence of IBS among adults is 10–20% (2), and 9.5–25% of people experience IBS symptoms (3). In Korea, the prevalence of IBS is 3–27% and is higher in women than in men (4). Furthermore, 20–50% of patients who registered in the digestive medicine department of a national tertiary medical institution were treated for IBS symptoms, and the prevalence of those affected was found to be increasing by 1% annually (5).

Most intestinal disorders are related to the environment, food, inflammation, and gut microbiota alterations (6). Although the pathophysiology of IBS is unclear, factors such as stress, abnormal bowel movements, altered brain-gut axis, gut microbiota changes, and psychological factors are known to be involved (7). In addition, low levels of inflammation or immune activation in gut can occur in IBS (8), and inflammatory activation is closely related to gut hypersensitivity and epithelial dysfunction. Thus, it appears that relations between alterations in gut microbiota and inflammation play important roles in the pathology of IBS.

Kimchi is a traditional fermented vegetable food in Korea. Baechu kimchi is the most common type of kimchi, and its main ingredient is Baechu cabbage (*Brassica pekinensis* Rupr.). As ingredients such as radish, cucumber, fish, and fruits can also be used to produce kimchi, there are many different types. Kimchi contains vitamin C, minerals, phytochemicals, dietary fiber, and lactic acid bacteria (LABs) (9). Kimchi can be eaten raw, but the fermented type is far more commonly consumed. Kimchi is spontaneously fermented at low temperatures (2–5°C), and plant-derived LABs (mainly *Lactobacillus plantarum, Leuconostoc mesenteroides*, and *Weissella koreensis*) are responsible for this fermentation (10). Kimchi has various beneficial functionalities such as antioxidant (11), anti-obesity (12, 13), antidiabetic (14), and anticancer (9) effects. In general, kimchi has beneficial effects on the human intestine, though these effects can be enhanced by optimizing fermentation conditions and ingredients. We developed a functional kimchi (FK) by adding functional sub-ingredients with anti-colitis (15) or anti-colorectal cancer (16) activities that enhance human colon health and improve metabolic parameters (17). In addition, FK has been reported to have anti-obesity (18) effects.

Here, we investigated and compared the effects of two new types of FK, developed by ourselves, and dead nano-sized *Lactobacillus plantarum* nF1 (nLp) added to standard kimchi (SK) with normal (standard) kimchi on individuals with IBS symptoms.

## Materials and methods

### Trial design

This study was designed to confirm the beneficial effect of kimchi with increased functionality on the symptoms of IBS. We were unable to compose a control group (a non-kimchi intake group) because almost all Koreans eat kimchi of some type daily, and thus, the predicted compliance throughout the 12-week intervention period was expected to be unacceptably low. Therefore, we composed three kimchi intake groups (standard kimchi, SK; nLp added standard kimchi, nLpSK; FK). The study subjects were asked to eat 210 g of kimchi daily (70 g per meal) and maintain their regular eating habits but not to consume probiotics. Subjects visited Kosin University Gospel Hospital (Busan, South Korea) at study commencement (baseline) and on the final day of the experiment (at the end of week 12). Anthropometric data, blood samples, and fecal samples were collected at baseline and at study completion. In addition, 3-day dietary records and IBS symptom questionnaires were also collected at these times.

The study was conducted in accordance with the Declaration of Helsinki, and the study protocol was approved by the Institutional Review Board of Kosin University College of Medicine (Busan, South Korea; approval number: 1040549-201409-BM-014). An informed consent was obtained from all study subjects, and consent for fecal sample collection was obtained from subjects who agreed to provide samples.

### Subjects

Subjects were recruited by advertising on the Pusan National University homepage and noticeboards in the Kosin University Gospel Hospital. Ninety subjects with IBS symptoms (aged 19–75) were recruited. Eligible subjects with symptoms meeting the Rome III criteria (19) for a diagnosis of IBS were recruited. Subjects were classified into three IBS subtypes: constipation-predominant (IBS-C), diarrhea-predominant (IBS-D), or mixed symptoms of constipation and diarrhea (IBS-M). Group allocations were randomized using a randomization program in SAS system (SAS Institute, Inc., Cary, North Carolina, USA). The exclusion criteria applied were as follows: a disease like to influence study findings, consistent intake of drugs affecting GI function (antispasmodics, antidiarrheals, laxatives, drugs that improve alimentary tract movement, tranquilizers, and dietary fiber supplements) within the 2 weeks preceding study commencement, pregnancy, or more than twice the normal level of creatinine, aspartate aminotransferase (AST), or alanine aminotransferase (ALT).

**Fig. 1:**
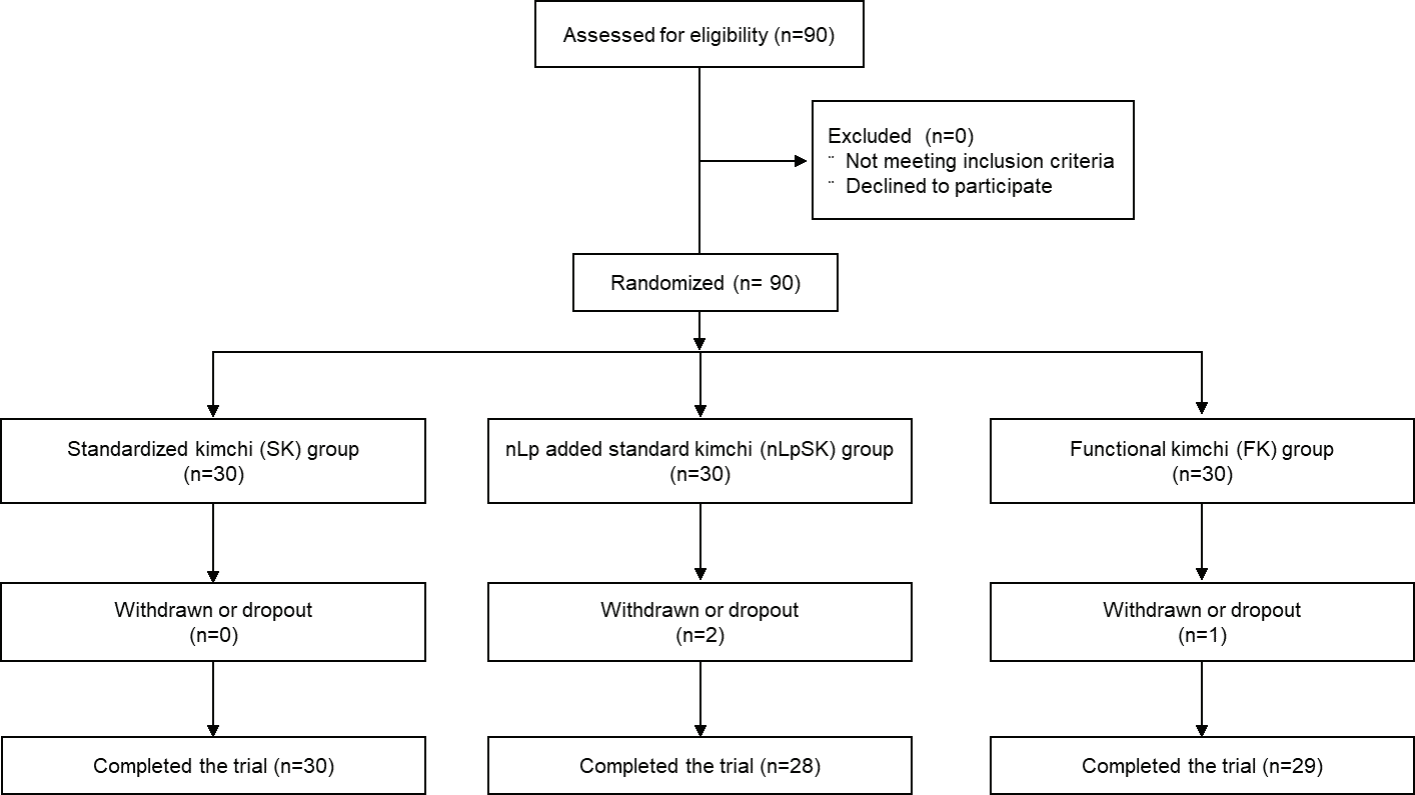
Randomization and follow-up flowchart.

To select a valid number of test subjects, we referred to other study similar to the present study (20) and calculated. Superiority testing was used to calculate the minimum number of subjects per group required (22 subjects), assuming a dropout percentage of 25. As a result, 90 subjects (17 males and 73 females) were enrolled (30 subjects/group) and double-blind randomized to the three groups. Two subjects (one in the nLpSK group and one in the FK group) dropped out during first week, and one subject in the nLpSK group during sixth week. Thus, 87 subjects (SK, *n* = 30; nLpSK, *n* = 28; FK, *n* = 29) completed the clinical study (Fig. 1). No subject consumed any other medicine or probiotic prior to or during this clinical study. Base characteristics of subjects are presented in Table 1.

**Table 1:** Baseline characteristics of subjects

Characteristics	SK	nLpSK	FK	*P*
*N*	30	28	29	
Sex (male/female)	3/27	9/21	5/25	0.131
Age	43 ± 10	40 ± 11	42 ± 13	0.836
Height (cm)	159.2 ± 8.4	161.3 ± 8.5	161.3 ± 5.8	0.499
Weight (kg)	57.5 ± 11.4	58.2 ± 9.5	57.5 ± 8.7	0.949
Type of IBS				0.424
IBS-D	3	2	2	
IBS-C	3	6	9	
IBS-M	24	22	19	

Results are presented as mean ± SDs. Analyzed using the Chi-square (χ^2^) test. SK, standard kimchi group; nLpSK, dead nano-sized *Lactobacillus plantarum* nF1 (nLp) added to standard kimchi group; FK, functional kimchi group; IBS, irritable bowel syndrome; IBS-D, diarrhea-predominant IBS; IBS-C, constipation-predominant IBS; IBS-M, mixed symptoms of constipation and diarrhea of IBS.

### Kimchi preparation

Three types of kimchi were made using standardized recipes (Table 2) (17) and packed (210 g/pack) by Hankaram Food Co., Ltd. (Ulsan, South Korea). Dead nano-sized *Lactobacillus plantarum* nF1 (nLp) deposited at the NITE Biological Resource Center (Tokyo, Japan; Accession No. NITE- P1462) was provided by Biogenics Korea Co., Ltd. (Seoul, South Korea). *Lactobacillus plantarum* PNU was isolated from well-fermented kimchi in our laboratory and deposited at the Korean Culture Center of Microorganisms (Registration No. KCTC 11352P), and powdered *Lactobacillus plantarum* PNU was provided by Ambio Co. (Jinju, South Korea). Korean mistletoe extract was purchased from Mistle Biotech Co. (Pohang, South Korea), and other ingredients were purchased from a local market in Busan, South Korea.

**Table 2:** Kimchi recipes for standard kimchi (SK), dead nano-sized *Lactobacillus plantarum* nF1 (nLp) added to standard kimchi (nLpSK), and functional kimchi (FK)

Ingredients	SK (g)	nLpSK (g)	FK (g)
Brined Baechu cabbage	100.0	100.0	100.0
Red pepper powder	3.5	3.5	2.5
Crushed garlic	1.4	1.4	2.8
Crushed ginger	0.6	0.6	0.6
Anchovy juice	2.2	2.2	-
Radish	13.0	13.0	11.0
Green onion	2.0	2.0	2.0
Sugar	1.0	1.0	1.0
Mustard leaf	-	-	7.5
Chinese pepper	-	-	0.1
Pear	-	-	2.8
Mushroom and sea tangle juice	-	-	5.0
Mistletoe extract (%)	-	-	0.05
Dead nano-sized *Lactobacillus plantarum* nF1 (g/kg)	-	2.0	-
*Lactobacillus plantarum* PNU (CFU/g)	-	-	1 × 10^6^
Final salt concentration (%)	2.2	2.2	2.2

The packages of kimchi were delivered to subjects by parcel delivery once per fortnight for 3 months. Subjects were instructed to keep the kimchi samples at 1 to 5°C in a normal or kimchi refrigerator and to consume one pack per day.

### Food intake records

Food intake records of the previous 3 days (2 weekdays and a day of weekend) were collected for the first and 12th weeks. Consumed nutrients were analyzed using Can-pro, a diet analysis program (The Korean Nutrition Society, Seoul, South Korea).

### Anthropometric measurements and serum analysis

Anthropometric measurements and blood analysis were conducted at baseline and at study completion. Heights and bodyweights were measured using an anthropometer and a scale. Body mass indices (BMIs, kg/m^2^) were calculated by dividing weight by height squared. Blood was collected from subjects in a 10-h fasted state. Inflammatory factors in serum, that is, interleukin (IL)-4, IL-6, IL-8, IL-10, IL-12, tumor necrosis factor (TNF)-α, interferon (IFN)-γ, and monocyte chemotactic protein (MCP)-1, were automatically analyzed using Quantikine ELISA Human lmmunossay kits (R&D Systems Inc., MN, USA) and an automatic microplate reader (Molecular Devices, USA) at the Seegene Medical Foundation (Seoul, South Korea).

### IBS symptom analysis

Completed IBS symptom evaluation questionnaires were collected at baseline and study completion. The questionnaire required subjects to detail IBS symptoms over the 3 days before visiting hospital.

- Irritable bowel symptoms: The four IBS symptoms, that is, abdominal pain or inconvenience, desperation, incomplete evacuation, and bloating, were each evaluated using a 5-point Likert scale (1 = no symptom; 2 = not very severe; 3 = quite severe; 4 = severe; 5 = very severe).
- Changes in defecation times during the previous 3 days and the last stool type were evaluated. Last stool type was evaluated using the Bristol stool scale (type 1 = separate hard lumps; type 2 = lumpy and sausage-like; type 3 = a sausage shape with surface cracks; type 4 = smooth, soft sausage, or snake-like; type 5 = soft blobs with clear-cut edges; type 6 = mushy consistency with ragged edges; and type 7 = liquid consistency with no solid pieces) (21).
- Improvements in overall irritable bowel symptoms: At study completion, subjects evaluated their overall IBS symptoms with respect to symptoms at baseline using a 5-point Likert scale (1 = much better; 2 = better; 3 = unchanged; 4 = worse; 5 = much worse).

### Fecal enzyme activity analysis

Fecal enzyme activities were analyzed using a slight modification of a previously described method (22). Initially, 1 g of stool was homogenized with 4 mL of 0.1M sodium phosphate buffer and centrifuged (13,000 rpm, 30 min).

- To determine β-glucosidase activity, a homogenized fecal sample was reacted with 10 μM para-nitrophenyl-β-D-glucoside for 5 min at 45°C, then 0.5M Na_2_CO_3_ was added, and the sample was centrifuged (4,000 × g). Supernatant absorbance was measured at 400 nm.
- To determine β-glucuronidase activity, a homogenized fecal sample was reacted with 10 μM phenolphthalein-β-D-glucuronic acid for 15 min at 45°C, and a 0.2M glycine-NaOH buffer was then added and centrifuged (4,000 × g). Supernatant absorbance was measured at 540 nm.

### Analysis of fecal microbiota using a pyrosequencing method

DNA was extracted using a FastDNA Spin Kit (Qiagen, Carlsbad, CA, USA) from homogenized fecal samples (*n* = 3 in per group). Extracted metagenomic DNA was then subjected to pyrosequencing (23). Briefly, polymerase chain reaction (PCR) was performed using barcoded fusion primers (http://www.ezbiocloud.net/resource/M1001) in a C1000 Touch thermal cycler (Bio-Rad, Foster, CA, USA). PCR reaction mixes contained 100 ng of template DNA, 0.2 mM dNTP, 0.5 μM primer, Ex Taq buffer, and two units of Ex Taq (all from Takara, Otsu, Japan). After initial denaturation (95°C for 5 min), amplification was performed over 30 amplification cycles (denaturation [95°C for 30 s], annealing [55°C for 30 s], and extension [72°C for 30 s]) followed by a final elongation (72°C for 5 min).

PCR products were subjected to electrophoresis and purified using QIAquick PCR purification kits (Qiagen, Valencia, CA, USA). After pooling purified products, short fragments of non-targeted products were eliminated. Product sizes and qualities were analyzed using a 2100 Bioanalyzer (Agilent, Palo Alto, CA, USA) and a DNA 7500 chip. Mixed amplicons were subjected to emulsion PCR. Sequencing was performed using the GS Junior Sequencing system (Roche, Branford, CT, USA) in Chunlab, Inc. (Seoul, South Korea). Pyrosequencing analysis was conducted as previously described (24, 25). The taxonomic classification of reads was conducted using the EzTaxon-e database (26).

### Statistical analysis

Categorical data were analyzed using the Chi-square (χ^2^) test. Data changes between baseline and study completion (end of week 12) were compared using the paired *t*-test. For microbiome analysis, the paired *t*-test was used to analyze groups nLpSK (*n* = 3) and FK (*n* = 3); however, the SK group was not included in the statistical analysis due to a lack of valid fecal samples (*n* = 2). The analysis was performed using SPSS statistics Ver. 18 (IBM Co., Armonk, NY, USA). Results are presented as mean ± standard deviations (SDs), and statistical significance was accepted for *P*-values <0.05.

## Results

### Nutrients and experimental kimchi intakes

Mean amounts of kimchi consumed before study commencement were 56.5 ± 39.7 g/day in the SK group, 54.1 ± 37.7 g/day in the nLpSK group, and 58.0 ± 45.8 g/day in the FK group. Random assignments created no problem as no significant difference was observed between the three groups. Intakes of energy and nutrients, and experimental kimchi consumptions were determined using food intake records (Table 3). In all groups, energy, fiber, and consumptions of three major nutrients (carbohydrate, protein, and fat) were greater at study completion than at baseline. Groups nLpSK and FK showed significant increases in carbohydrate consumption, and group SK showed a significant increase in protein and fat consumptions. All three groups showed a significant increase in fiber intake (*P* < 0.05 in the SK and nLpSK groups and *P* < 0.01 in the FK group). The percentages of experimental kimchi consumed in all groups ranged from 85 to 90. BMIs were unchanged in all groups during the experimental period.

**Table 3:** Changes in nutrient and experimental kimchi intakes and body mass index (BMI)

	Baseline			Week 12		
	SK	nLpSK	FK	SK	nLpSK	FK
Energy (kcal)	1508.7 ± 341.2	1500.0 ± 387.8	1516.9 ± 394.3	1781.6 ± 519.6	1761.3 ± 381.4*	1796.4 ± 415.7*
Carbohydrate (g)	252.2 ± 68.7	226.9 ± 64.4	237.2 ± 57.2	254.7 ± 73.8	276.3 ± 74.4*	295.8 ± 79.2*
Protein (g)	55.8 ± 20.8	65.7 ± 31.1	57.9 ± 26.0	76.2 ± 29.6*	72.7 ± 31.0	69.3 ± 19.9
Fat (g)	32.1 ± 12.0	39.3 ± 23.1	39.2 ± 29.2	54.2 ± 27.0**	44.5 ± 15.1	40.3 ± 14.4
Fiber (g)	19.1 ± 6.5	17.9 ± 6.4	17.4 ± 6.1	25.2 ± 10.2*	24.4 ± 9.7*	23.0 ± 8.0**
Kimchi consumption rate (%)	-	-	-	90.4 ± 11.7	90.1 ± 10.3	85.2 ± 19.8
BMI (kg/m^2^)	22.5 ± 2.7	22.3 ± 2.0	22.0 ± 2.6	22.4 ± 2.4	22.3 ± 1.8	22.0 ± 2.4

Results are presented as mean ± SDs. *P < 0.05 and **P < 0.01 versus baseline.

SK, standard kimchi group (*n* = 30); nLpSK, dead nano-sized *Lactobacillus plantarum* nF1 (nLp) added to standard kimchi group (*n* = 28); FK, functional kimchi group (*n* = 29).

### IBS symptoms

IBS symptoms and overall improvements were analyzed using questionnaire responses and 5-point Likert and Bristol stool scales (Table 4). The four IBS symptoms (abdominal pain or inconvenience, desperation, incomplete evacuation, and bloating) were significantly improved in all groups (*P* < 0.001). Group defecation frequencies all adjusted to around 3.0 times/3 days (*P* < 0.05). As regards stool types, subjects in the SK, who had small lumpy stools at baseline, had normal sausage or snake-like stools at study completion (from 2.6 ± 1.8 to 4.0 ± 0.4, *P* < 0.001, as determined by Bristol stool scale). Subjects in the FK group also showed significant improvement (from 3.5 ± 1.5 to 3.9 ± 1.2, *P* < 0.01). Subjects in all groups scored overall improvement in IBS symptoms as ‘good’ (score 2).

**Table 4:** Changes in irritable bowel syndrome symptom scores

IBS symptoms	Baseline			Week 12		
	SK	nLpSK	FK	SK	nLpSK	FK
Abdominal pain or inconvenience	2.4 ± 0.6	2.4 ± 0.5	2.5 ± 0.6	1.4 ± 0.5***	1.6 ± 0.5***	1.4 ± 0.4***
Desperation	2.2 ± 0.9	2.6 ± 0.6	2.2 ± 0.8	1.3 ± 0.5***	1.3 ± 0.4***	1.4 ± 0.5***
Incomplete evacuation	2.5 ± 0.8	2.6 ± 0.8	2.4 ± 0.8	1.4 ± 0.5***	1.5 ± 0.5***	1.4 ± 0.6***
Bloating	2.6 ± 0.7	2.7 ± 0.7	2.5 ± 0.6	1.3 ± 0.5***	1.5 ± 0.6***	1.4 ± 0.6***
Defecation times	2.6 ± 1.7	3.4 ± 2.0	2.5 ± 1.4	2.9 ± 1.1*	3.0 ± 1.2*	2.8 ± 1.1*
Last stool type	2.6 ± 1.8	3.6 ± 1.5	3.5 ± 1.5	4.0 ± 0.4***	3.9 ± 0.5	3.9 ± 1.2**
Improvement of overall IBS symptoms	-	-	-	2.0 ± 0.6	2.0 ± 0.5	2.3 ± 0.6

Results are presented as mean ± SDs. *P < 0.05, **P < 0.01, and ***P < 0.001 versus baseline.

SK, standard kimchi group (*n* = 30); nLpSK, dead nano-sized *Lactobacillus plantarum* nF1 (nLp) added to standard kimchi group (*n* = 28); FK, functional kimchi group (*n* = 29).

### Inflammatory factors in serum

Changes in serum cytokine levels over the study period are detailed in Table 5. Subjects in all three groups showed a significant decrease in TNF-α (*P* < 0.001) at study completion. The nLpSK and FK groups showed significant decreases in IL-4 (*P* < 0.001), IL-10 (*P* < 0.001), and IL-12 (*P* < 0.01), and the nLpSK had a significantly lower level of MCP-1 (*P* < 0.05). IL-8 levels were fell non-significantly in all groups over the study period.

**Table 5:** Changes in serum inflammatory factors

Inflammatory factors	Baseline			Week 12		
	SK	nLpSK	FK	SK	nLpSK	FK
TNF-α (pg/mL)	6.5 ± 2.3	6.7 ± 2.2	6.4 ± 1.7	3.3 ± 2.7***	3.3 ± 1.5***	2.9 ± 1.6***
IL-4 (pg/mL)	14.0 ± 1.5	14.2 ± 1.5	13.7 ± 1.2	13.2 ± 2.1	12.7 ± 1.0***	12.3 ± 1.3***
IL-6 (pg/mL)	3.7 ± 0.9	4.1 ± 1.5	4.2 ± 1.3	4.2 ± 0.8	4.0 ± 0.7	4.1 ± 0.6
IL-8 (pg/mL)	99.5 ± 65.8	123.8 ± 116.6	144.2 ± 134.0	95.9 ± 108.8	105.4 ± 98.6	105.9 ± 86.2
IL-10 (pg/mL)	5.9 ± 2.5	7.0 ± 2.6	5.6 ± 1.5	4.8 ± 5.1	4.8 ± 2.5***	4.0 ± 1.8***
IL-12 (pg/mL)	6.0 ± 0.8	6.0 ± 0.7	5.9 ± 0.7	5.6 ± 1.0	5.4 ± 0.6**	5.2 ± 1.0**
IFN-γ (pg/mL)	12.1 ± 2.7	12.5 ± 4.1	12.0 ± 1.6	13.2 ± 2.1	12.7 ± 1.8	12.1 ± 1.4
MCP-1 (pg/mL)	206.8 ± 53.1	252.2 ± 80.3	207.4 ± 62.1	202.0 ± 41.9	214.5 ± 53.7*	189.0 ± 47

Results are presented as mean ± SDs. *P < 0.05, **P < 0.01, and ***P < 0.001 versus baseline.

SK, standard kimchi group (*n* = 30); nLpSK, dead nano-sized *Lactobacillus plantarum* nF1 (nLp) added to standard kimchi group (*n* = 28); FK, functional kimchi group (*n* = 29).

### Fecal enzyme activities and microbiome analyses

**Fig. 2:**
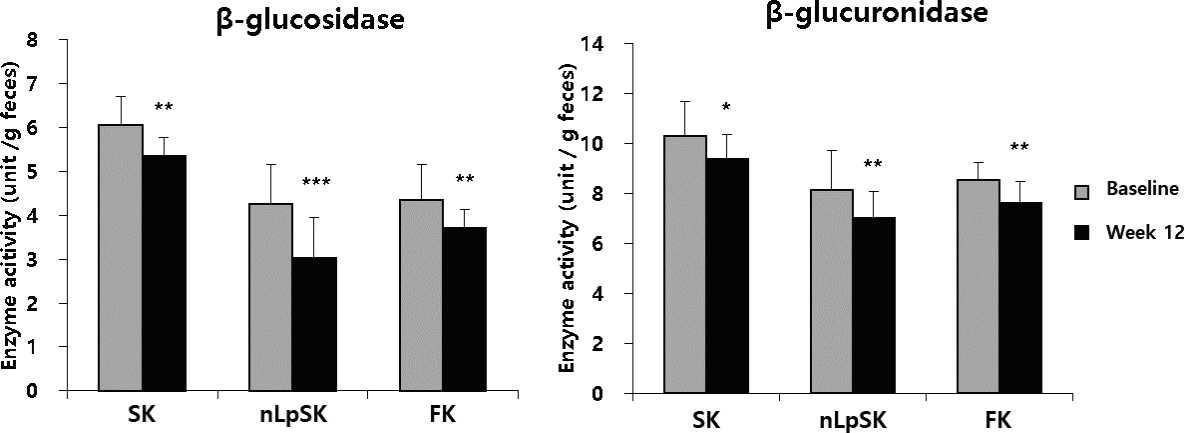
Fecal β-glucosidase and β-glucuronidase activity changes. Results are presented as means ± SDs. **P < 0.01 and ***P < 0.001 versus baseline. SK, standard kimchi group (*n* = 5); nLpSK, dead nano-sized *Lactobacillus plantarum* nF1 (nLp) added to standard kimchi group (*n* = 6); FK, functional kimchi group (*n* = 6).

The activities of β-glucosidase and β-glucuronidase, two harmful fecal enzymes, fell in all three groups during the 12-week study period (Fig. 2). β-Glucosidase activity fell in the nLpSK group from 4.26 ± 0.90 units/g feces to 3.04 ± 0.90 unit/g (*P* < 0.001), in the SK group from 6.06 ± 0.64 to 5.35 ± 0.43 units/g (*P* < 0.01), and in the FK group from 4.35 ± 0.82 to 3.71 ± 0.42 units/g (*P* < 0.01). β-Glucuronidase activity fell in the nLpSK group from 8.13 ± 1.20 to 7.02 ± 0.99 units/g (*P* < 0.01), in the FK group from 8.55 ± 0.53 to 7.62 ± 0.67 units/g (*P* < 0.01), and in the SK group from 10.33 ± 0.61 to 9.39 ± 0.08 units/g (*P* < 0.05).

**Fig. 3:**
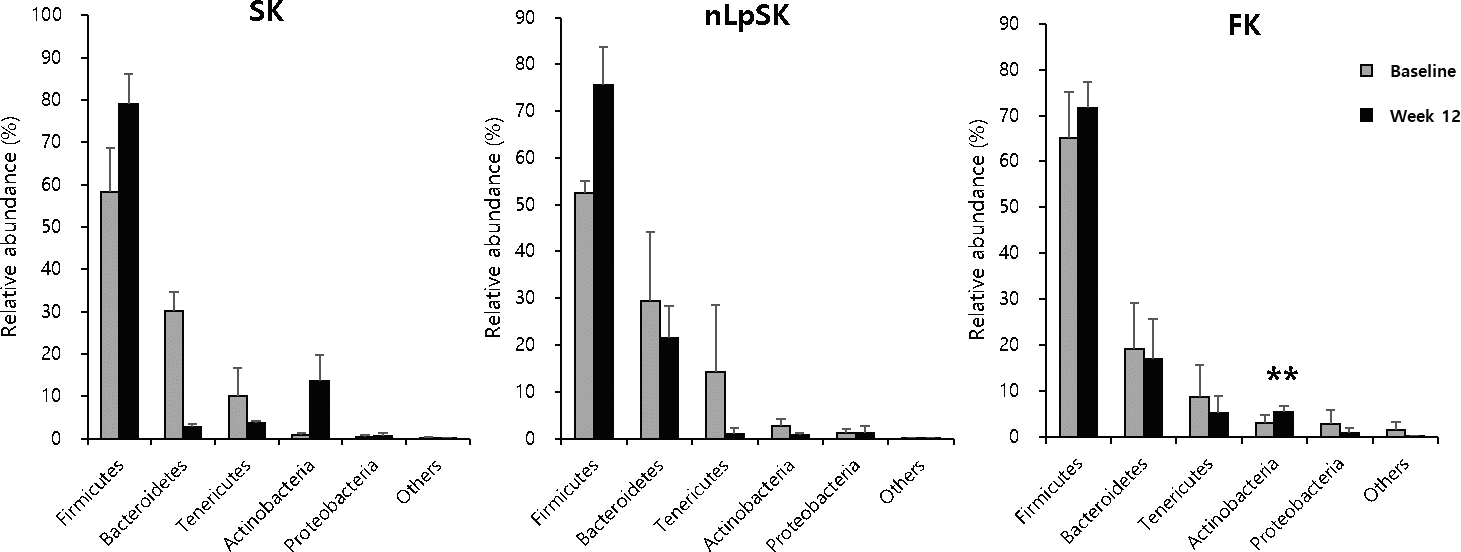
Composition changes in fecal microbiomes at the phylum level. Results are presented as means ± SDs. **P < 0.01 and ***P < 0.001 versus baseline. SK, standard kimchi group (*n* = 2); nLpSK, dead nano-sized *Lactobacillus plantarum* nF1 (nLp) added to standard kimchi group (*n* = 3); FK, functional kimchi group (*n* = 3).

Fecal samples for microbiome analysis were collected from nine subjects (three subjects from each group), but unfortunately, DNA extraction from the sample of one subject in the SK group failed. Therefore, we analyzed changes in the samples of three subjects in the nLpSK and FK groups and two subjects in the SK group. All three groups showed increases in Firmicutes and decreases in Bacteroidetes and Tenericutes. In particular, Actinobacteria in the FK group (*P* < 0.01) were significantly higher at study completion (Fig. 3).

**Fig. 4:**
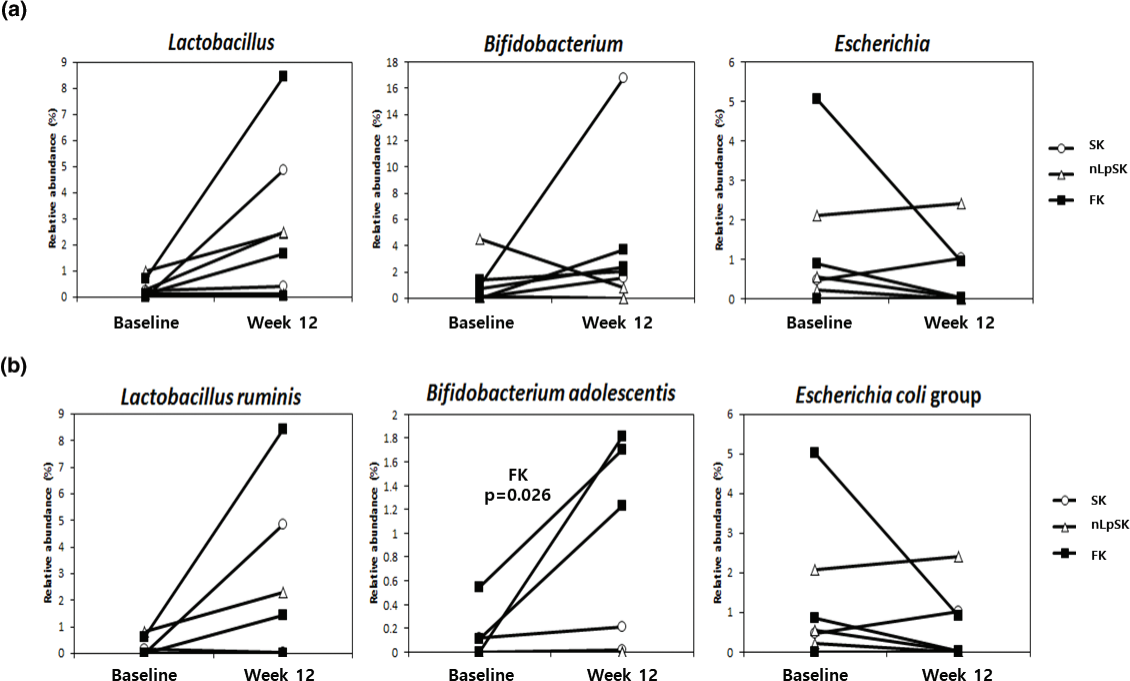
Composition changes in fecal microbiomes at the genus (a) and species (b) levels. Results are presented as means ± SDs. *P*-values were determined using the paired *t*-test. SK, standard kimchi group (*n* = 2); nLpSK, dead nano-sized *Lactobacillus plantarum* nF1 (nLp) added to standard kimchi group (*n* = 3); FK, functional kimchi group (*n* = 3).

At the genus level, most subjects showed increases in probiotic genera *Lactobacillus* and *Bifidobacterium* populations and decreases in *Escherichia* (Fig. 4a). Recently, the genus *Lactobacillus* was reclassified. Zheng et al. (27) classified that the genus *Latobacillus* in the present study includes *Lactobacillus delbrueckii* group, *Paralactobacillus*, and other 23 novel genera such as *Holzapfelia, Amylolactobacillus, Bombilactobacillus*, etc.

At the species level, several subjects showed increased levels of *Lactobacillus ruminis* and *Bifidobacterium adolescentis* (Fig. 4b). Notably, all subjects in the FK group showed significant increases in *Bifidobacterium adolescentis* (*P* = 0.026). ‘*Escherichia coli* group’ includes six pathogenic species (*Escherichia coli, Escherichia albertii, Shigella flexneri, Shigella sonnei, Shigella boydii,* and *Shigella dysenteriae*; http://www.ezbiocloud.net/eztaxon/taxonomic_group) with similar 16S rRNA sequences. Almost all subjects had a lower *Escherichia coli* group population at study completion, except two subjects in the nLpSK group.

## Discussion

Kimchi is a widely consumed traditional Korean fermented vegetable-based food. We attempted to improve the functionalities of kimchi by controlling fermentation conditions and adding sub-ingredients, and in this study, we investigated the beneficial health effects of the three types of kimchi (SK, nLpSK, and FK) *in vitro, in vivo,* and in a clinical study of their effects on the symptoms of IBS (12, 15–18, 28). Our results confirm the beneficial effects of kimchi on these symptoms.

According to several studies, young adults in Korea consume 33.7–48.3 g of kimchi per day (17, 29), whereas in the present study, subjects consumed 54–58 g/day. We suspect this difference was caused by different age ranges, as subjects enrolled in the present study were considerably older (age range: 19–75). Older people consume more dietary fiber, and kimchi is the main source of dietary fiber for Korean adults over the age of 20 (30). In the present study, energy, carbohydrate, protein, fat, and dietary fiber intakes increased in all three groups during the study period, presumably because of increased consumptions of foods other than kimchi. In particular, the significant increases in dietary fiber intakes observed may have been due to increased kimchi consumptions. In previous studies, significant increases in energy consumption but no changes in body weight or BMI were considered to indicate kimchi has an anti-obesity effect (12, 13, 17), and in a previous clinical study, food intakes were increased by the consumptions of SK or FK for 4 weeks, but body weights were not increased (17). Therefore, it appears that any type of kimchi can help reduce body weight and BMI.

Inflammation and cytokine imbalance in the presence of mental stress and abnormal gut condition are probably important etiologic factors of IBS (31). IBS is sometimes described as a mild chronic inflammation caused by elevated pro-inflammatory cytokine levels, and many studies have reported that IBS patients have significantly higher serum levels of pro-inflammatory cytokines (e.g. TNF-α, IL-1β, IL-6, IL-12, and IFN-γ) and chemokines (e.g. IL-8 and MCP-1) than healthy controls (31–33). Circulating TNF-α and IL-6 is associated with IBS symptoms of abdominal pain and discomfort (34), and TNF-α is associated with anxiety, depression, and fatigue in IBS (34). Our observation that subjects in all three study groups had significantly lower TNF-α levels at study completion (*P* < 0.001, Table 5) seems to be related to observed improvements in abdominal pain and abdominal inconvenience (*P* < 0.001, Table 4). IL-12 is related to mild chronic inflammation, and MCP-1 assembles monocytes and macrophages at inflammatory sites in many inflammatory conditions (32). We observed subjects in groups nLpSK and FK exhibited significant reductions in serum IL-12 levels at study completion (*P* < 0.01 for both). In addition, MCP-1 levels were significantly reduced in the nLpSK group (*P* < 0.05). These findings indicate that the functional ingredients in nLpSK and FK helped reduce pro-inflammatory factors in IBS subjects. Th2 cytokines are a family of anti-inflammatory cytokines, and IL-4 and IL-10 are representative of these cytokines. Tendency of the level of IL-4 and IL-10 in blood (plasma, serum, or peripheral blood mononuclear cells [PBMC]) in IBS patients is not yet clear (34). Few studies have investigated IL-4 changes in serum, though IL-4 levels have been reported to be high in the colon mucosa of IBS patients (35). In addition, several studies have reported elevated IL-10 levels in the PBMCs of IBS patients (33, 36, 37) because elevated circulatory IL-10 levels in IBD patients are attributed to inflammatory reaction of colitis (38). In the present study, serum levels of IL-4 and IL-10 were lower in the nLpSK and FK groups at study completion (*P* < 0.001 per group, Table 5), which suggests that the functional ingredients in nLpSK and FK, such as nLp, *Lactobacillus plantarum* PNU, mistletoe extract, and mustard leaves, helped to control immune functions.

nLp was the only sub-ingredient in nLpSK. nLp (size 0.5–1.0 µm) is a shrunken processed form of kimchi-derived *Lactobacillus plantarum* nF1 and is manufactured by incubating *Lactobacillus plantarum* nF1 under harsh conditions (40°C, 1.0% (w/w) salinity, and pH 5.0), and following this with high-pressure homogenization to produce a nano-dispersion (39). Small sized-LABs have previously been found to increase immune activity by controlling IL-12 and IFN-α cytokine secretions (40) and increasing antigen uptake by Peyer’s patches (41). We previously reported that in a mouse model of colon cancer, nLp treatment increased the fecal secretion of IgA as compared with normally sized live *Lactobacillus plantarum* nF1-treated mice (28). In addition, kimchi with added nLp and nLp reduced dextran sulfate sodium (DSS)-induced colitis (15) and azoxymethane/DSS-induced colon cancer (28) in mice.

FK contains several sub-ingredients with known anti-inflammatory effects. Mistletoe has immune response controlling (42) and anticancer (43) effects, and mustard leaves have anti-inflammatory, antidiarrheal, anticancer, and antioxidant effects (44, 45). We confirmed the anti-inflammatory effect of FK in our previous studies (16, 17).

Probiotic LABs such as *Lactobacillus rhamnosus* GG, *Propionibacterium freudenreichii, Bifidobacterium animalis* (46), and *Bifidobacterium infantis* 35624 (47) alleviate IBS symptoms. Also, the genera *Bifidobacterium, Lactobacillus, Streptococcus* improve abdominal pain, abdominal distension, and gas production (48). In addition, probiotic LABs and dietary fiber improve constipation by reducing bowel movements and shortening stool passage time (49, 50). Similarly, the intake of kimchi, which is rich in fiber and LABs, can improve IBS symptoms. *Lactobacillus plantarum* PNU, which was used as a starter in FK, has strong probiotic effects, which include antioxidative, intestinal adherent activity, thermal stability, resistance to gastric acid and bile acid, and anticancer effects (51), and thus, perhaps enhanced gut functions in the FK group.

The red pepper powder produced in Korea that is used to make kimchi contains about 10–30 mg of capsaicin (the ‘hot’ component of red pepper) per 100 g (52). Usually, Korean adults consume capsaicin from red pepper powder at around 10–60 mg per day (52), and capsaicin is 82 to 89% absorbed in the GI tract 3 h after consumption (53, 54). The concentration of capsaicin in the test kimchis used in this study was not measured, but assuming 30 mg per 100 g of red pepper powder, the daily intakes of SK and nLpSK (210 g) contained 1.8 mg of capsaicin and FK contained 1.2 mg. Sometimes, hot red pepper powder can irritate the digestive system, but when administered to patients with functional dyspepsia, red pepper powder improved symptoms of indigestion (upper abdominal pain and nausea) (55). In our study, IBS symptoms were improved in all subjects, and no subject reported indigestion or stomach problems caused by eating kimchi.

β-Glucosidase can produce toxic aglycones from plant glycosides, and β-glucuronidase hydrolyzes glucuronic acid conjugates and increases the enterohepatic circulation of toxic substances (56). Also, β-glucuronidase converts pre-carcinogens into carcinogens (57). These harmful enzymes have been reported to be less active in Koreans and Germans that eat fermented vegetables such as kimchi or sauerkraut (58). In this study, all subjects showed lower levels of β-glucosidase and β-glucuronidase activities at study completion (Fig. 2). Therefore, our observations suggest kimchi consumption can reduce the formation of intestinal toxins and carcinogens by lowering the activities of these harmful enzymes in the intestinal tract.

In the gut microbiome of IBS patients, the Firmicutes to Bacteroidetes ratio and the population of Proteobacteria (59, 60) were increased, but the population of Actinobacteria was decreased (59). Also in IBS patients, *Streptococcus*, *Ruminococcus, Dorea*, and *Clostridium* were found to be increased (60), while *Lactobacillus, Bifidobacterium*, and *Faecalibacterium* were decreased (60–62). In our study, firmicutes populations were increased after intervention. Furthermore, actinobacteria counts were higher, and tenericutes and proteobacteria counts were lower in most samples (Fig. 3). Although not significant, probiotic genera *Lactobacillus* and *Bifidobacterium* populations were higher at study completion than at baseline in all groups (Fig. 4a). These findings demonstrate that kimchi intake can change the gut microbiome and increase probiotic genera populations. Naturally fermented well-ripened kimchi (around pH 4.3) contains 10^8-9^ CFU/g of LABs. The experimental FK kimchi may have contained more than 10^8-9^ CFU/g LAB because live *Lactobacillus plantarum* PNU (10^6^ CFU/kg kimchi) was used as a starter. However, few amount of kimchi LABs (some strains in *Lactobacillus*, *Weissella*, and *Leuconostoc*) were identified in the subject’s feces, which means that the kimchi LABs did not settle in the subject’s intestines. Unexpectedly, the *Bifidobacterium adolescentis* proportion was significantly higher in the FK group at study completion (*P* < 0.05). This observation is in line with our previous clinical study (17), in which healthy individuals who consumed FK showed an increase in the *Bifidobacterium adolescentis* proportion after 4 weeks of kimchi intake. Thus, it would appear that *Lactobacillus plantarum* PNU and naturally formed *Lactobacillus plantarum* in kimchi, rather than dominating bacteria in gut, promote the establishment of other LABs, such as *Bifidobacterium adolescentis.* Interestingly, pathogenic *Escherichia coli* group species counts were reduced by intervention in most subjects (Fig. 4b), which suggested *Lactobacillus plantarum* protected individuals from *Escherichia coli* infections (17).

## Conclusion

This study demonstrates the beneficial effects of kimchis (SK, nLpSK, and FK) in IBS subjects. Dietary fiber intake increased significantly in the SK, nLpSK, and FK groups over the intervention period, and IBS symptoms (abdominal pain or inconvenience, desperation, incomplete evacuation, and bloating) and stool form improved. Furthermore, subjects in all groups were satisfied with the way kimchi consumption improved IBS symptoms. Of the three types of kimchi investigated, nLpSK most effectively controlled immunity and inhibited harmful intestinal enzyme activity, and interestingly, FK enhanced the growth of *Bifidobacterium adolescentis* in gut. Thus, this study shows that kimchi can improve the symptoms of IBS, and that adding sub-ingredients can enhance its effects.
